# Predicting surgical outcome in posterior retroperitoneoscopic adrenalectomy with the aid of a preoperative nomogram

**DOI:** 10.1007/s00464-021-09005-9

**Published:** 2022-01-13

**Authors:** Allon van Uitert, Elle C. J. van de Wiel, Jordache Ramjith, Jaap Deinum, Henri J. L. M. Timmers, J. Alfred Witjes, Leo J. Schultze Kool, Johan F. Langenhuijsen

**Affiliations:** 1 Department of Urology, Radboud University Medical Center, P.O. Box 9101, 6500 HB Nijmegen, The Netherlands; 2 Department of Health Evidence, Radboud University Medical Center, P.O. Box 9101, 6500 HB Nijmegen, The Netherlands; 3 Department of Internal Medicine, Radboud University Medical Center, P.O. Box 9101, 6500 HB Nijmegen, The Netherlands; 4 Department of Radiology and Nuclear Medicine, Radboud University Medical Center, P.O. Box 9101, 6500 HB Nijmegen, The Netherlands

**Keywords:** Adrenalectomy, Nomogram, Retroperitoneoscopic, Laparoscopy, Predictive model, Operative time

## Abstract

**Background:**

Posterior retroperitoneoscopic adrenalectomy (PRA) has several advantages over transperitoneal laparoscopic adrenalectomy (TLA) regarding operative time, blood loss, postoperative pain, and recovery. However, it can be a technically challenging procedure. To improve patient selection for PRA, we developed a preoperative nomogram to predict operative time.

**Methods:**

All consecutive patients with tumors of ≤ 7 cm and a body mass index (BMI) of < 35 kg/m^2^ undergoing unilateral PRA between February 2011 and March 2020 were included in the study. The primary outcome was operative time as surrogate endpoint for surgical complexity. Using ten patient variables, an optimal prediction model was created, with a best subsets regression analysis to find the best one-variable up to the best seven-variable model.

**Results:**

In total 215 patients were included, with a mean age of 52 years and mean tumor size of 2.4 cm. After best subsets regression analysis, a four-variable nomogram was selected and calibrated. This model included sex, pheochromocytoma, BMI, and perinephric fat, which were all individually significant predictors. This model showed an ideal balance between predictive power and applicability, with an *R*^2^ of 38.6.

**Conclusions:**

A four-variable nomogram was developed to predict operative time in PRA, which can aid the surgeon to preoperatively identify suitable patients for PRA. If the nomogram predicts longer operative time and therefore a more complex operation, TLA should be considered as an alternative approach since it provides a larger working space. Also, the nomogram can be used for training purposes to select patients with favorable characteristics when learning this surgical approach.

The first transperitoneal laparoscopic adrenalectomy (TLA) was described by Gagner et al. in 1992 [1]. The posterior retroperitoneoscopic adrenalectomy (PRA) was first introduced in 1994 [2]. The technique of PRA was modified by Walz who introduced several preoperative selection criteria, such as body mass index (BMI) of < 35 kg/m^2^, tumor size ≤ 7 cm, and low suspicion of malignancy [3]. In various studies both techniques were compared with the open approach, and proved to be safe and effective with low morbidity and complication rates, decreased blood loss, less postoperative pain, shorter hospital stay, and improved cosmetics [4, 5]. Currently, minimally invasive adrenalectomy is the standard of care for the management of benign adrenal tumors and, in selected cases, for the treatment of small (≤ 6 cm) malignant tumors [6]. In a randomized controlled trial, Barczynski et al. showed several advantages for PRA over TLA, including shorter operative time, less blood loss, less postoperative pain, faster recovery, improved cost-effectiveness, and abolished risk of trocar site herniation due to the direct approach to the adrenal gland avoiding intra-abdominal dissection and manipulation [7]. We previously showed that the median operative time in our hospital is 90 min for unilateral TLA and 57 min for unilateral PRA [8]. Especially, in bilateral cases, PRA shows significant advantages regarding blood loss and operative time, since there is no need to reposition the patient [9]. Although PRA is getting more and more popular, it can be technically challenging due to the smaller working space and the paucity of anatomical landmarks that exist “en route”. Therefore, we believe that in some cases TLA is the better approach, but patient selection remains challenging.

Several studies showed that a higher BMI results in longer operative time [10–12]. Furthermore, patient characteristics such as sex, histopathology, tumor side, and various radiological measurements were correlated with longer operative time, but did not result in higher complication rates [13–16]. However, the results of these studies vary, patient numbers are small, and no tool to predict operative time is currently available from literature. Therefore, in this study, we aimed to develop a preoperative nomogram for predicting operative time of PRA, using both patient characteristics and radiological measurements of retroperitoneal fat including the anatomical position of the adrenal gland. Since the number of complications and conversion rates are generally low, operative time was used as surrogate endpoint for surgical complexity. If the nomogram predicts longer operative time and therefore a more complex operation, TLA may be considered as an alternative approach.

## Materials and methods

### Data collection

In 2011, we introduced PRA in our hospital for selected patients because of its potential advantages. All consecutive patients who underwent unilateral PRA between February 2011 and March 2020 were included in this study and every procedure was performed by one expert urologist (JL), who was trained by Walz in the operative technique [16].

Patients were eligible for PRA with a BMI of < 35 kg/m^2^, a tumor size ≤ 7 cm, and low suspicion of malignancy, including pheochromocytomas [6]. Other indications were primary aldosteronism, Cushing syndrome, atypical nonfunctioning adenoma, extra-adrenal paraganglioma located cranially of the renal vessels, and a history of extensive intra-abdominal surgery. If patients did not meet these selection criteria, TLA or open adrenalectomy was performed. The study was approved by the local Medical Ethics Committee, who waived the need to obtain informed consent since patients were not subjected to investigational actions. Patient confidentiality was guaranteed according to the Dutch law on personal data protection.

**Fig. 1:**
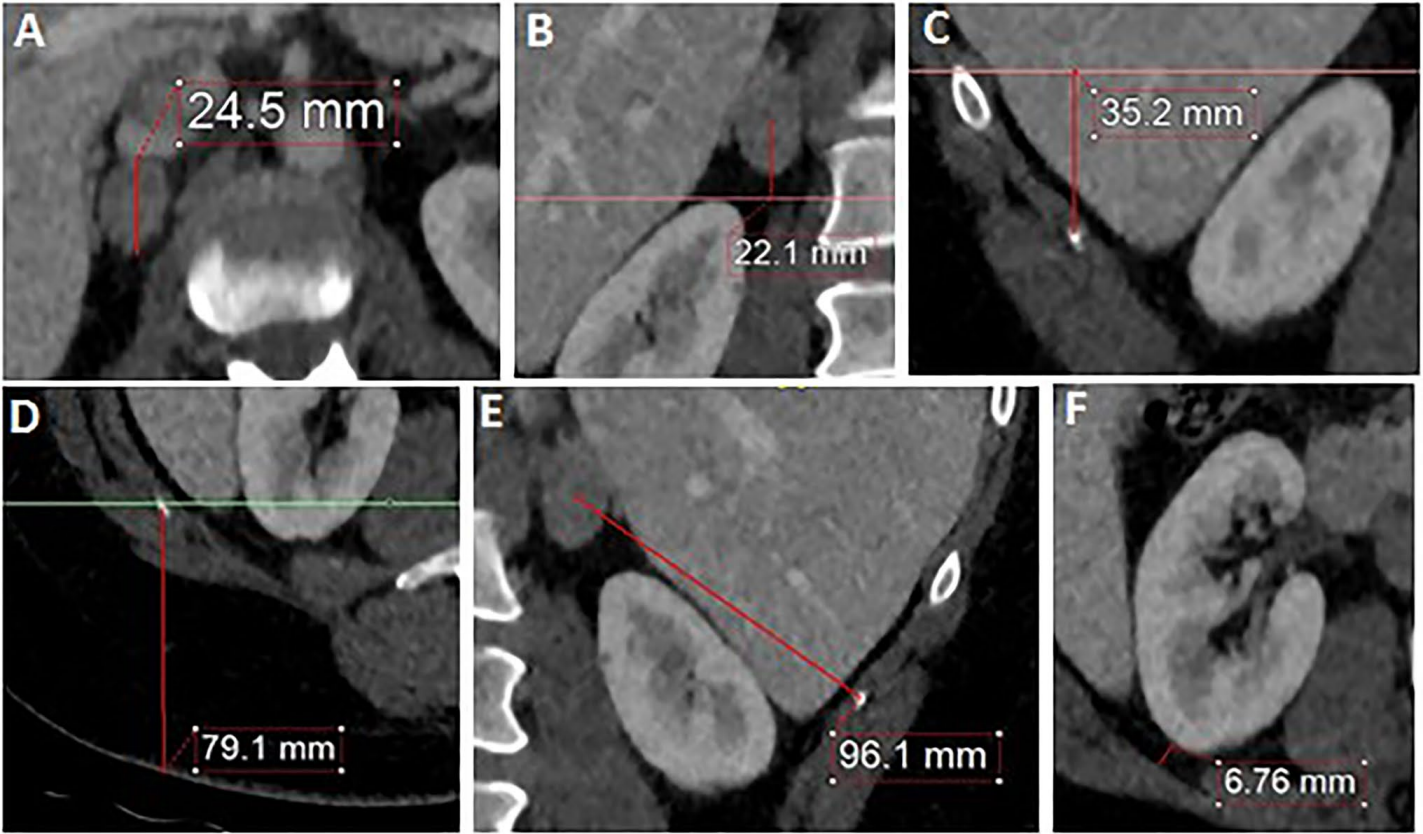
Pictures illustrating the radiological measurements on abdominal CT. **A** tumor size. **B** vertical distance tumor. **C** position of the kidney. **D** skin distance. **E** adrenal depth. **F** perinephric fat

The primary outcome variable was skin-to-skin operative time. The predicting variables included sex, BMI, tumor side, indication for surgery, and six quantitative radiological measures (Fig. 1). All perioperative data were prospectively collected in a database, including patient characteristics, operative time, blood loss, conversions, and perioperative complications.

### Radiological measurements

Six quantitative radiological measurements were performed on preoperative CT-scans using Aquarius iNtuition Viewer version 4.4.12 (TeraRecon Inc., Durham, NC, USA). Two researchers (AU and EW) were trained to perform these measurements by an expert radiologist (LS). The first twenty patients were excluded from the study for practicing reasons to increase reproducibility of the measurements by both researchers, and to correct for the learning curve for PRA, which is estimated to be between 20 and 40 procedures according to the literature [8, 17]. Thereafter, the radiological measurements were performed separately, but for every tenth patient the same measurement was done by both researchers to calculate interrater reliability.

The tip of the 12th rib and the upper pole of the kidney were used as radiological landmarks, since these are easy to localize on preoperative CT-scans. Furthermore, these are important surgical landmarks for PRA, since the first trocar is placed at the tip of the 12th rib and the surgical route to the adrenal gland is made by mobilizing the upper pole of the kidney. When patients had a rudimentary 12th rib (< 5 cm length), the tip of the 11th rib was used as landmark. First, tumor size was defined as the largest diameter of the mass in the axial plane (Fig. 1a). The height of the tumor was defined as the vertical distance between the center of the tumor and the upper margin of the kidney in the coronal plane (Fig. 1b). The position of the upper pole was defined as the vertical distance from the upper margin of the kidney to the tip of the 12th rib in the coronal plane (Fig. 1c). The skin distance was defined as the vertical distance from the tip of the 12th rib to the skin in the axial plane (Fig. 1d). Adrenal depth was defined as the distance between the center of the tumor and the tip of the 12th rib, which was measured as the shortest distance in an oblique plane showing both structures (Fig. 1e). Perinephric fat was defined as the perpendicular distance of the kidney to the abdominal wall, measured at the height of the renal hilum in the axial plane (Fig. 1f).

### Nomogram development

For development of the nomogram, we used operative time as the primary outcome. First, we applied a stepwise selection procedure for multivariable linear regression analysis to find the model that fitted the data best, with respect to Akaike’s Information Criterion [18, 19]. Subsequently, we performed a best subsets regression analysis to find the best one-variable model up to the best seven-variable model [19, 20]. Then, we identified a smaller model with optimized balance between predictive power and practicality by utilizing fewer variables with an adequate prediction performance. We calculated the adjusted *R*^2^ and mean square error to indicate the predictive performance of the model. To calibrate the final model, we plotted predicted log duration versus observed log duration. To facilitate use of the model, an online application was created, which calculates the predicted operative time and provides a 95% prediction interval for each combination of variables as well.

For all analyses, we used RStudio, version 3.6.2 (R Foundation for statistical computing, Vienna, Austria) [21].

## Results

### Baseline characteristics and radiological measurements

In total, 215 patients were included in the study. Patient demographics are summarized in Table 1. The mean age was 51.6 ± 12.2 years and 49% were male. Mean tumor size was 2.4 ± 1.6 cm. Two patients had an extra-adrenal paraganglioma, both of which were located cranially of the renal vessels, directly adjacent to the adrenal gland. After reviewing all preoperative CT-scans, 91% of the radiological data points were measurable, which were included in the analysis. Since there was a high correlation between BMI and waist circumference (*R*^2^ = 0.789), BMI was included in the model since this is usually more readily available. Radiological measurement outcomes are summarized in Table 2.

**Table 1:** Summary of patient demographics and surgical outcomes

Patient characteristics	Total *n* = 215
Sex (*n*)	
Male	105 (48.8)
Female	110 (51.2)
Age (years)	51.6 ± 12.2
BMI (kg/m^2^)	26.5 ± 3.8
< 18.5	3 (1.4)
18.5–25	87 (40.4)
25–30	90 (41.9)
30–35	33 (15.3)
> 35	2 (9.3)
Clinical diagnosis (*n*)	
Pheochromocytoma	52 (24.2)
Primary aldosteronism	140 (65.1)
Cushing syndrome	16 (7.4)
Other	7 (3.2)
Side (*n*)	
Right	101 (47)
Left	114 (53)
Operating time (min)	68.4 (31.5)
Blood loss (mL)	5 (5–10)
Conversions (*n*)	11 (5,1)
Duration of admission (days)	3 (3–4)
Complications (Clavien Dindo), (*n*)	
Grade 0	198 (92.1)
Grade 1	9 (4.2)
Grade 2	6 (2.8)
Grade 3	1 (0.5)
Grade 4	1 (0.5)

Categorical variables are presented as *n* (%)

Continuous variables are presented as mean ± standard deviation if normally distributed, otherwise as median (interquartile range)

**Table 2:** Radiological measurements

Tumor size (mm)	23.7 ± 15.5
Adrenal—kidney (mm)	1.1 (− 10.5 to 9.0)
12th rib—kidney (mm)	− 49.4 ± 23.9
12th rib—skin (mm)	44.9 (37.9–54.1)
12th rib—adrenal (mm)	95.7 ± 17.4
Perinephric fat (mm)	11.5 (5.6–18.9)

Variables are presented as mean ± standard deviation if normally distributed, otherwise as median (interquartile range)

Negative values are defined in reference to the upper pole of the kidney

*Mm* millimeter

### Interrater reliability

We used the measurements of 21 patients to calculate interrater reliability between the two researchers. The interrater reliability was 0.991 [95% confidence interval (CI) 0.979–0.996] for tumor—upper pole distance, 0.998 (95% CI 0.995–0.999) for upper pole—12th rib distance, 0.993 (95% CI 0.982–0.997) for skin—12th rib distance, 0.993 (95% CI 0.983–0.997) for tumor—12th rib distance, and 0.974 (95% CI 0.897–0.991) for perinephric fat. Tumor size was already available from the CT-scan reports.

### Selecting the final nomogram

**Fig. 2:**
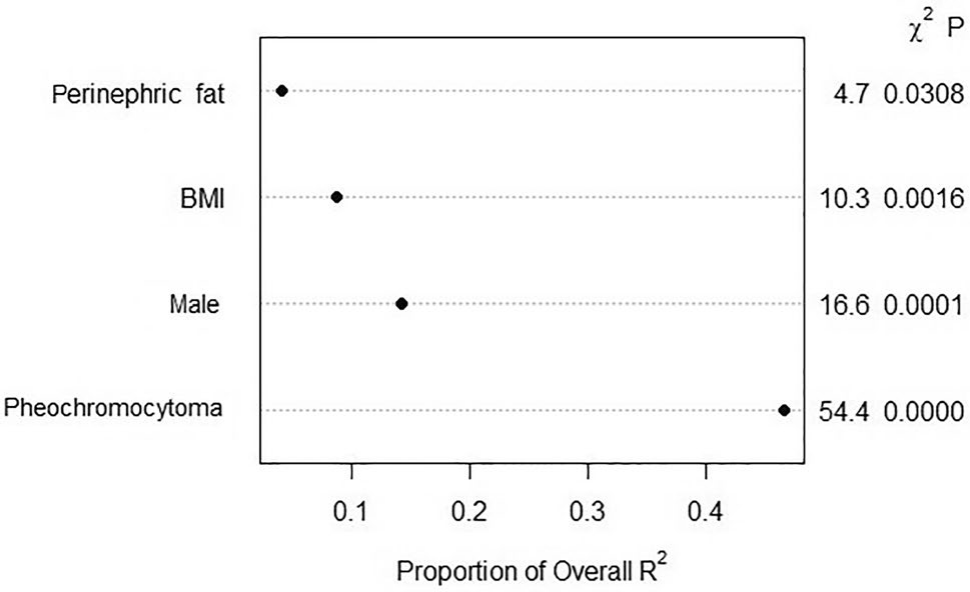
Individual weight of the four variables in the final model

Since operative time showed a right-skewed distribution, log transformation was performed in order to satisfy the criteria of normality. The stepwise selection procedure showed that the seven-variable model fitted the data best with an *R*^2^ of 40.2 (Table 3). After the best subsets regression analysis, we identified seven different models ranging from the best one-variable model up to the best seven-variable model (Table 3). The one-variable model had an *R*^2^ of 15.8, the seven-variable model had an *R*^2^ of 40.2. The seven-variable model explained 1.6% more of the variation in operative time than the four-variable model, which was not statistically significant. Furthermore, in the four-variable model, all individual variables were significant predictors (Fig. 2). Therefore, we chose the four-variable model as the final model because it showed the best balance between predictive power and applicability, with an *R*^2^ of 38.6. Pheochromocytoma was the most important predictive factor in this model for longer operative time.

**Table 3:**
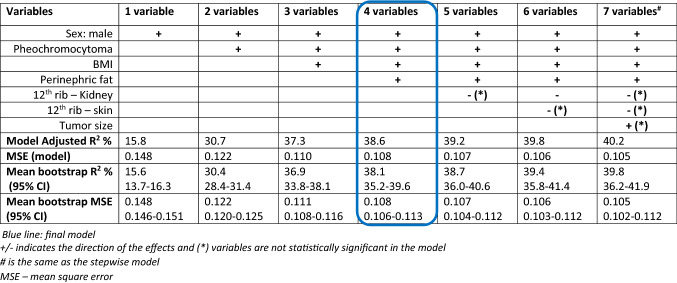
Predictive performance of the variables

**Fig. 3:**
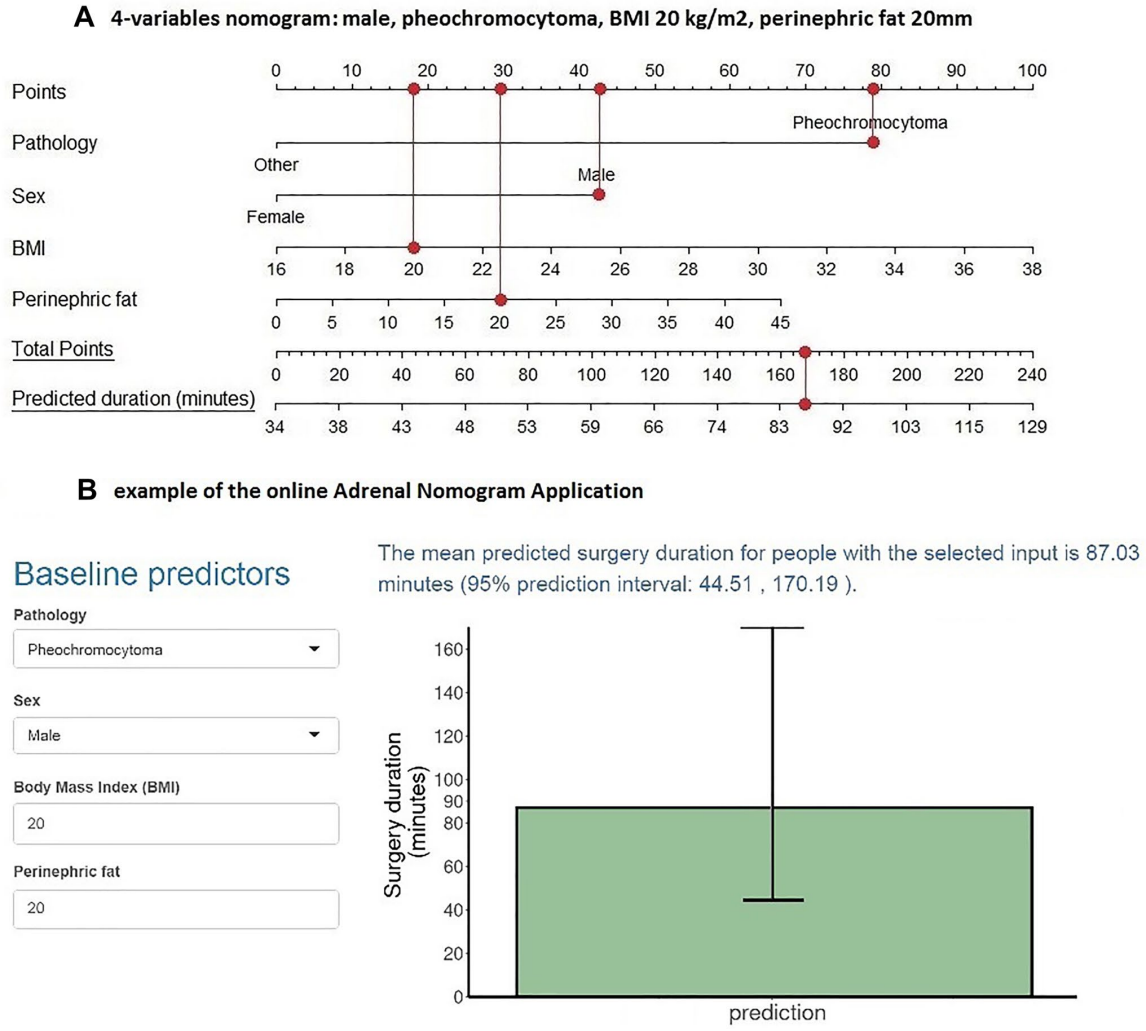
Application of the nomogram

**Fig. 4:**
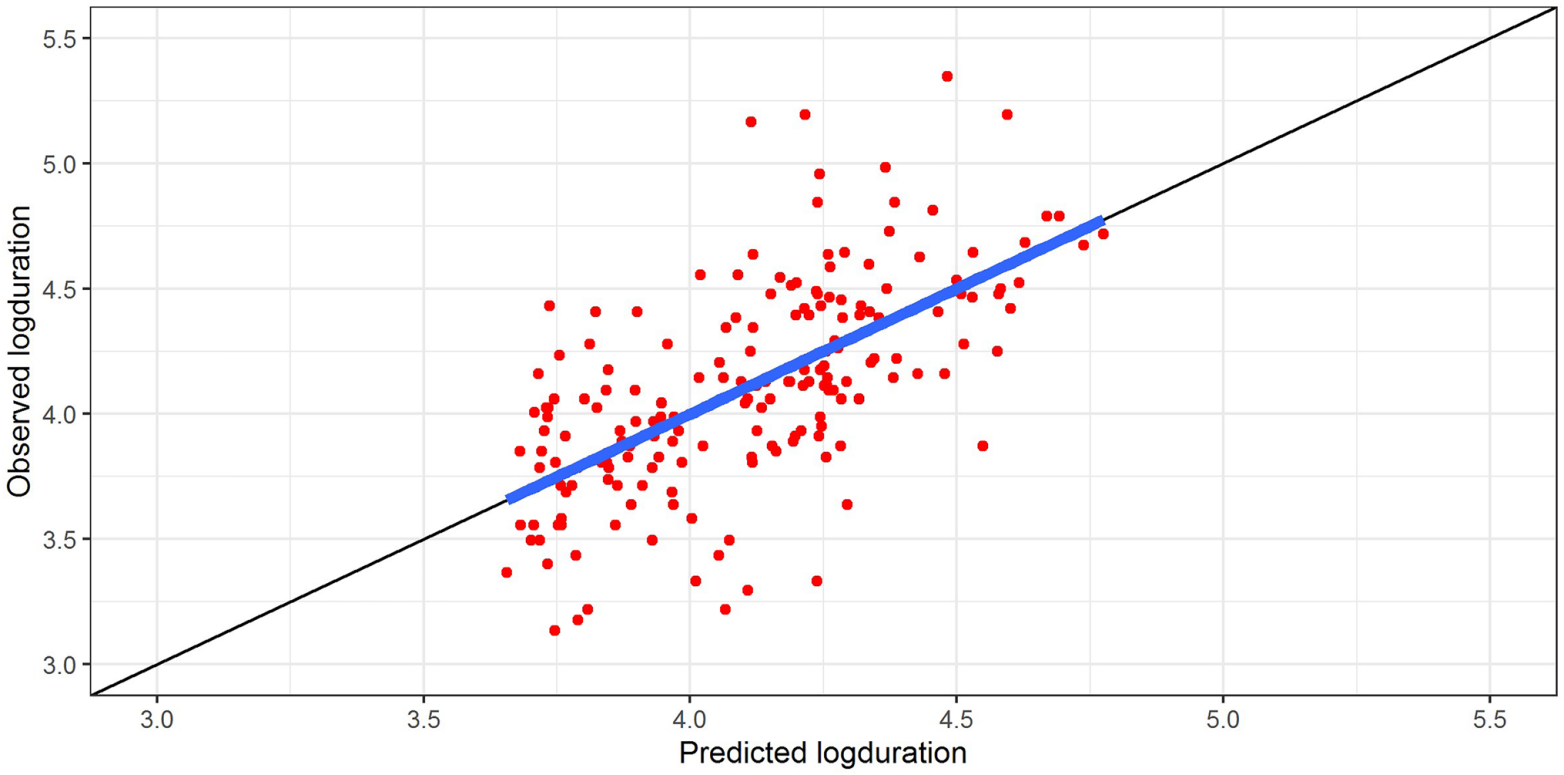
Calibration curves compare predicted and observed log duration. The blue line shows that the slope for the relationship between the predicted log duration and the observed log duration was 1.00 (95% CI 0.82–1.18) and the intercept was 0 (95% CI − 0.75 to 0.75)

An example of applying the model is shown in Fig. 3a: a male patient (43 points) with a BMI of 20 (18 points), a pheochromocytoma (78 points), and perinephric fat of 20 mm (29 points) scores 168 points. In this patient, the nomogram predicts an operative time of 87 min. An example of applying the model using the online application is shown in Fig. 3b. This model is available to use online [22]. The predicted and observed log duration are shown in Fig. 4, showing a well-calibrated four-variable model.

### Conversions

In ten patients (4.7%) conversion to TLA was necessary due to insufficient working space and in one patient to open surgery due to bleeding of the caval vein. When comparing baseline characteristics of this group with the rest of the patients, sex, BMI, side, adrenal depth, and perinephric fat were significantly different (Table 4). Furthermore, the mean predicted operative time in this group was significantly higher (79 min vs. 64 min, respectively).

**Table 4:** Baseline characteristics of converted versus nonconverted patients

	Converted *n* = 11	Not converted *n* = 204	*P* value
Age during surgery (years)	49.7 ± 10.6	51.7 ± 12.4	0.60
Sex (male)	9 (81.8)	96 (47.1)	0.025
BMI (kg/m^2^)	31.0 ± 4.0	26.3 ± 3.6	0.000
Operating time (min)	147.5 (28.6)	64.2 (25.5)	0.000
Blood loss (mL)	523.2 (1334.8)	13.4 (31.6)	0.23
Duration of admission (days)	5.7 (7.5)	3.2 (1.0)	0.28
Tumor size (mm)	18.7 (16.9)	23.9 (15.4)	0.28
Indication for adrenalectomy, *n* (%)			0.70
PA	7 (63.6)	133 (65.2)	
Pheochromocytoma	2 (18.2)	15 (7.4)	
M. Cushing	1 (9.1)	50 (24.5)	
Other	1 (9.1)	6 (2.9)	
Side of adrenalectomy (left/right); *n* (%)	1 (9.1)/10 (90.9)	113 (55.4)/91 (44.6)	0.003
Radiological measurements			
Height of tumor (mm)	5.4 (14.3)	− 1.1 (14.9)	0.17
Height of kidney (mm)	− 59.3 (16.4)	− 48.8 (24.1)	0.16
Skin distance (mm)	50.4 (11.9)	46.9 (12.8)	0.45
Adrenal depth (mm)	110.5 (15.6)	94.8 (17.2)	0.003
Perinephric fat (mm)	20.4 (7.9)	12.3 (8.4)	0.002
Length of 12th rib (mm)	125.3 (17.9)	109.1 (27.5)	0.06
Predicted operating time (min)	78.7 (15.7)	63.5 (17.9)	0.006

Categorical variables are presented as *n* (%)

Continuous variables are presented as mean ± standard deviation

## Discussion

In this study, we developed a preoperative nomogram to predict operative time using a large cohort of patients who underwent PRA. There are several advantages of PRA when compared to TLA, such as shorter operative time, less blood loss, less postoperative pain, faster recovery, improved cost-effectiveness, abolished risk of trocar site herniation due to the direct approach to the adrenal gland, less risk for damaging intra-abdominal organs, and shorter length of stay [7]. However, it can be challenging due to the smaller working space and paucity of anatomical landmarks compared to TLA, and in case of emergency conversion to open surgery there is a need to reposition the patient, possibly causing unwanted delay. Meanwhile, TLA is a well-known approach to most laparoscopic surgeons, and urgent conversion to open surgery is straightforward.

This nomogram was developed to preoperatively aid the surgeon in selecting the best approach for each individual patient. Although several studies correlated different parameters with operative time, to the best of our knowledge, a predictive nomogram has not been developed before. Agcoaglu et al. analyzed several parameters and operative time in 82 patients who underwent either TLA or PRA [12]. In their study, a preoperative selection between both techniques was made based on anthropomorphic parameters. Operative time was correlated with BMI in TLA and with perinephric fat thickness and the distance between the adrenal tumor and the upper pole of the kidney in PRA. However, the number of patients included in both study arms was small and no selection algorithm was described. Lindeman et al. investigated 113 patients who underwent either PRA or TLA [14]. This study showed a strong correlation of the “Posterior Adiposity Index” (sum of distances of skin-to-Gerota and perinephric fat) with operative time in PRA. After performing multivariable regression analyses, right-sided tumors, tumor size, and the “Posterior Adiposity Index” were significant predictors of operative time. However, only 56 patients in this group received PRA, patients who underwent TLA had a significantly higher BMI, and the operations were performed by four different surgeons. Pearlstein et al. retrospectively investigated the influence of anthropomorphic measurements on operative time in 83 patients receiving PRA [15]. In their study, a multivariable linear random effects model was developed, which showed that periadrenal fat volume, surgeons experience, and a right-sided operation were significant predictors of operative time.

In our four-variable nomogram, pheochromocytoma was the strongest predictor for operative time. This can be explained by the fact that pheochromocytomas consist of highly vascularized and fragile tissues, requiring careful dissection, resulting in longer operative time. The second predictor of operative time was male sex. This can be explained by more adherent perinephric fat to the renal capsule in males [23], which hampers mobilization of the upper pole of the kidney. Third, BMI was a significant predictor of operative time, which can be explained by the subsequent limited working space. Fourth, perinephric fat was significantly correlated with operative time, which also hampers freeing of the kidney. Although there is a clear relation between perinephric fat and BMI, both factors individually contributed significantly to the model.

Considering the ten patients in whom conversion to TLA was necessary, several baseline characteristics of these patients were unfavorable for an uncomplicated and fast operation and the predicted operative time was significantly higher compared to other PRA patients.

To select the optimal surgical approach for paragangliomas, the localization of the tumor is the most important factor. Paragangliomas that are located cranially of the renal vessels (upper retroperitoneum) can be operated by the posterior retroperitoneal approach. Paragangliomas below the renal vessels (lower retroperitoneum) should preferably be operated by the transperitoneal lateral approach. This is in accordance to the literature by Choi et al. [24] and Walz et al. [25, 26]. In all cases, we would recommend performing these procedures in an expert center, since these tumors are usually very adherent to the major vessels and well vascularized.

Working with a dedicated surgical team is a very important factor influencing operative time in minimally invasive surgery [27]. In our study, one urologist performed all procedures with a dedicated team of operating room nurses with a specific focus for minimally invasive surgery. Therefore, we did not use a competency assessment tool, since the team composition was comparable between all procedures. However, the use of competency assessment tools to assess surgical expertise is recommended if different surgeons perform these procedures for the sake of pooling the results.

There are several strengths of our study. A large sample size was used for building this nomogram. One expert urologist performed all procedures, with a dedicated operating team with a focus on minimally invasive surgery. The first twenty patients were excluded for practicing reasons to increase reproducibility of the measurements between both investigators and to correct for the learning curve of our surgeon. The radiological measurements had a high interrater reliability after training by an expert radiologist. The model calibration showed a well-calibrated nomogram with an accurate model performance.

There are several limitations in this study. First of all, the final *R*^2^ of the model was 38.6, which means that almost 39% of the variation in operative time is reliably explained by this model. However, this also means that 61% of the variation in operative time is not explained by the model and is the result of other unknown factors. Therefore, a 95% prediction interval was provided to give a reliable range of the predicted operative time. Second, not every preoperative CT-scan was of sufficient quality to perform the radiological measurements, resulting in some missing data (9%). Third, all operations were performed by one surgeon, which interferes with extrapolation of the outcomes of this model to other surgeons. Fourth, operative time was the primary outcome as a surrogate endpoint for surgical complexity and it is debatable whether longer operative time as such, without complications, indicates an unprofitable outcome for the patient. However, this surgical endpoint is frequently used in literature as a surrogate for surgical complexity [13, 28], and it is the best available parameter for this type of surgery, due to the low number of conversions and complications.

In conclusion, a four-variable nomogram was developed to predict operative time in PRA. In our current strategy, a preoperative selection of patients for PRA is made based on tumor size and BMI. This nomogram can further aid the surgeon in this preselected group to make a choice for PRA in patients with an adrenal tumor. Although the 95% prediction interval is fairly wide, the model can indicate a complex procedure of PRA and since the online nomogram is easily accessible and easy to use, we think the nomogram is of added value as a clinical support tool to improve patient selection, surgical planning, and for training purposes. We believe TLA should be considered if the model predicts longer operative time and therefore a more complex operation, as it provides a larger working space. In general, we think that minimally invasive adrenal surgery may best be performed in high-volume expert centers with a proficiency in both techniques, enabling selection of the best technique for each individual patient. In training procedures, competency assessment could be supplementary to this nomogram, by not only selecting the right patient for the retroperitoneal approach, but also selecting the right surgeon for the right patient by adjusting for skill level of the surgeon. A prospective study by other expert centers would be beneficial to further validate our model.
